# Culturing Bacteria From Fermentation Pit Muds of Baijiu With Culturomics and Amplicon-Based Metagenomic Approaches

**DOI:** 10.3389/fmicb.2020.01223

**Published:** 2020-06-23

**Authors:** Jialiang Xu, Leping Sun, Xuan Xing, Zhanbin Sun, Haoyue Gu, Xin Lu, Zhenpeng Li, Qing Ren

**Affiliations:** ^1^School of Light Industry, Beijing Technology and Business University, Beijing, China; ^2^State Key Laboratory for Infectious Disease Prevention and Control, National Institute for Communicable Disease Control and Prevention, Chinese Center for Disease Control and Prevention, Beijing, China

**Keywords:** amplicon sequencing, culturomics, Baijiu, microbiota, pit mud

## Abstract

The Baijiu-making microbiota has an important role in the alcohol production, flavor, and character of Baijiu. 16S rRNA gene sequencing revolutionized the understanding of Baijiu-making microbiota. In this study, nine phyla, 23 classes, 49 orders, 99 families, and 201 genera were detected in pit muds (PMs) by 16S rRNA gene sequencing. Firmicutes and Bacteroidetes predominated (>99%). At the order level, Clostridiales, Bacteroidales, and Bacillales predominated (>92%). At the genus level, *Hydrogenispora*, *Petrimonas*, *Proteiniphilum*, and *Sedimentibacter* predominated. The pure culture of Baijiu-making prokaryotes was essential to elucidating the role of these microbes in the fermentation of Baijiu. According to the theory of microbial culturomics, a culturing approach with multiple culture conditions was adopted, combining 16S rRNA gene sequencing. We identified 215 prokaryotic strains, which were assigned to 66 species, 41 genera, four phyla, and 19 potential new species. Gas conditions were key factors in culturomics. In addition, culturomics significantly increased the number of species isolated from the fermentation PM compared with previous reports. With culturomics, the diversity spectrum of culturable bacteria in the PM was increased 273.33% at the genus level. This study confirms the complementary role of culturomics in the exploration of complex microbiota.

## Introduction

Baijiu is a well-known distilled spirit with a history of 2,000 years in China. Baijiu is a clear and transparent fermented alcoholic beverage. The fermentation process of Baijiu is usually performed in an underground fermentation pit (Liu and Sun, 2018). Pit mud (PM), a specific fermented clay, is a source of inoculum and a habitat of microbes in fermentation cellars for Baijiu production (Zheng et al., 2013; Tao et al., 2014). The PM hosts diverse microbial communities contributing to the production of characteristic flavor compounds (Liu et al., 2017a) that determine the flavor and quality of Baijiu.

Determining the microbial community of PM has always been a challenge. PM is an important business resource and must be domesticated for a long time (Chai et al., 2019). The advent of omics technologies has improved our knowledge of the Baijiu-making microbial ecosystem. In particular, the use of metagenomics or 16S rRNA gene sequencing has revealed the diversity of the Baijiu-making microbiota. Extensive studies have been performed to study the microbial communities of PM. These studies have shown that PM microbial communities are considerably complex (Zheng et al., 2013, 2015; Tao et al., 2014; Hu et al., 2016). A high diversity of approximately 16 phyla and 105 genera has been found in PM (Liu et al., 2017b). However, culture-independent approaches limit our understanding of the fermentation microecosystem and hinder the further exploration of microbial resources for biotechnological applications.

Culture-dependent approaches are indispensable for further studies of PM microbial function. In previous studies, a small number of bacterial species or genera were isolated, identified, and obtained from pure cultures. *Clostridium prazmowski* was reported as the main microorganism in the PM of *Wuliangye* Baijiu by using a culture-dependent method (Wu et al., 1991), whereas *Bacillus* and *Sporolactobacillus* spp. dominated in the PM of *Luzhoulaojiao* Baijiu (Yue et al., 2007). In addition, *Paenibacillus* (Chen et al., 2015), *Bacillus* (Huang et al., 2014; Ma et al., 2016), *Lysobacter* (Zhang et al., 2017), *Sporolactobacillus*, *Mycobacterium*, *Pseudomonas*, *Microbacterium*, *Corynebacterium*, *Flavobacterium* (Yue et al., 2007), *Mierococcus*, *Staphylococcus*, *Burkholderia* (Huang et al., 2014), *Brevibacillus*, and *Aneurinibacillus* spp. (He et al., 2016) were isolated from PM located in different places in previous studies. However, it is difficult to objectively reflect the actual microbial diversity and community composition in PM with these pure-culture microorganisms since only a few genera or species (only 15 genera) have been isolated. Furthermore, little is known about the genetic novelty of microbial communities, although many novel species have been isolated from PM (Liu C. et al., 2014; Chen et al., 2015; Ma et al., 2016; Yin et al., 2016). However, the discovery of multiple novel species suggests that PM might be a promising source of new taxa.

In recent years, culturomics, which uses multiple culture conditions combined with rapid identification, was developed to culture and identify unknown bacteria. In the first culturomics study, 212 culture conditions were used to generate more than 30,000 colonies, of which 341 bacterial species were cultured, including 31 new bacterial species (Lagier et al., 2012). Lagier et al. (2016) identified 1,057 prokaryotic species, thereby adding 531 species to the human gut repertoire, including 197 potentially new species, by culturomics. Hundreds of novel microorganisms related to the human microbiome have been cultured with culturomics (Lagier et al., 2018). However, culturomics has rarely been reported for the culture and identification of bacteria in PM.

In this work, we reported the bacterial diversity and richness of Baijiu-making microbiota from fermentation PM and applied culturomics to culture and identifying them. To our knowledge, this study systematically described the bacteria in PM for the first time with culturomics.

## Materials and Methods

### Samples

The Baijiu PM sample was collected from a Baijiu winery in Sichuan Province, China. The samples were sent to the laboratory located in Beijing on dry ice. The sample was divided into two parts: one part was used for culturing bacteria, and the other part was used for 16S rRNA gene sequencing. Triplicates were performed in this study.

### Microbial Diversity

Microbial DNA was extracted from PM using the M4015-01EZNA^®^ Soil DNA kit (Omega Bio-Tek, Norcross, GA, United States). The concentration and purity of DNA were determined by a NanoDrop 2000 UV-vis spectrophotometer (Thermo Scientific, Wilmington, United States). DNA quality was checked by 1% agarose gel electrophoresis. The V3–V4 hypervariable regions of the bacterial 16S rRNA gene were amplified by polymerase chain reactions (the reaction procedure was as follows: 95°C for 5 min; 25 cycles at 95°C for 30 s; 55°C for 30 s; 72°C for 40 s and 72°C for 10 min) with primers 338F (5′-ACTCCTACGGGAGGCAGCAG-3′) and 806R (5′-GGACTACHVGGGTWTCTAAT-3′) using a thermocycler PCR system (GeneAmp 9700, ABI, United States). PCR products were subjected to electrophoresis on a 2% agarose gel for detection. PCR products were purified and recovered by cutting the gel using the AxyPrep DNA Gel Recovery Kit (AXYGEN). Quantitative detection of PCR products was performed by the QuantiFluor^®^-ST Blue Fluorescence System (Promega). Sequencing libraries were generated using the TruSeq^®^ DNA PCR-Free Sample Preparation Kit (Illumina, United States). Purified amplicons were sent for Illumina high-throughput sequencing on a MiSeq platform (Illumina, CA, United States) (Caporaso et al., 2010; Xu et al., 2018). Raw fastq files were demultiplexed, quality-filtered by Trimmomatic, and merged by FLASH (Magoc and Salzberg, 2011) using the following criteria: (i) the reads were truncated at any site receiving an average quality score <20 over a 50 bp sliding window. (ii) Primers were exactly matched, allowing two nucleotide mismatches, and reads containing ambiguous bases were removed. (iii) Sequences whose overlap was longer than 10 bp were merged according to their overlap sequence. Sequences were clustered into operational taxonomic units (OTUs) at 97% sequence identity (Edgar, 2013). Chimeras were removed during the clustering process, which provided the representative sequence of each OTU (Edgar et al., 2011). The taxonomic assignment of OTUs from the phylum to genus level was performed using RDP (Usearch version 7.1)^1^ and the Silva reference database (Quast et al., 2013). Alpha diversity was applied to analyze the complexity of species diversity for a sample through the Shannon index and Simpson index. Indices were calculated by mothur (version v.1.30.1)^2^ (Schloss et al., 2011) and displayed with R software. The read sequences obtained from Illumina MiSeq were submitted to the NCBI Sequence Read Archive (SRA) under accession number SRR11624724.

### Culturomics

Culturomics is a high-throughput method that multiplies culture conditions in order to detect higher bacterial diversity and pure bacterial cultures (Lagier et al., 2012). This culturomics study included 27 culture conditions (including direct inoculation in various culture media) and anaerobic conditions (Table 1). The choice of medium has an extremely important effect on the culture of bacteria. The three groups of media included common commercial medium, original environmental medium, and predicted medium. The original environmental medium was 10 g of PM in 100 ml of deionized water with 20% (w/v) agar and 10 g of PM in 100 ml of deionized water with 10% (w/v) glucose and 20% (w/v) agar. The predicted medium was obtained from the Leibniz Institute DSMZ-German Collection of Microorganisms and Cell Cultures by KOMODO (Known Media Database) media recommendation system^3^ (Oberhardt et al., 2015) or from studies reporting on similar species. For example, *Hydrogenispora ethanolica* LX-B^T^ within the *Hydrogenispora* genus, which is predominant in PM, is the only type strain. It was not inhibited by ampicillin, chloramphenicol, streptomycin, or penicillin at 50 mg/L (Liu Y. et al., 2014). In order to isolate this rare genus, four antibiotics were added to exclude other genera that could not grow on these four antibiotics.

**TABLE 1:** Culture conditions used for culturing pit mud bacteria.

**No.**	**Medium**	**Gas**	**Additional reagent**
1	MRS	Aerobic	–
2	PDA	Aerobic	–
3	TSA	Aerobic	–
4	R2A	Aerobic	–
5	Pit muds	Aerobic	–
6	Pit muds	Aerobic	20 g/L Glucose
7	MRS	Microanaerobic	–
8	TSA	Microanaerobic	–
9	R2A	Microanaerobic	–
10	Lactic acid bacteria selective agar	Microanaerobic	–
11	Gaoshi No.1	Microanaerobic	–
12	R2A	Anaerobic	–
13	TSA	Anaerobic	–
14	Lactic acid bacteria selective agar	Anaerobic	–
15	Gaoshi No.1	Anaerobic	–
16	DSMZ-500	Anaerobic	–
17	DSMZ-500	Anaerobic	50 mg/L ampicillin
18	DSMZ-500	Anaerobic	50 mg/L pyrazosulfuron
19	DSMZ-500	Anaerobic	50 mg/L chloramphenicol
20	DSMZ-500	Anaerobic	50 mg/L penicillin
21	Optimum substrate agar	Anaerobic	–
22	Optimum substrate agar	Anaerobic	50 mg/L ampicillin
23	Optimum substrate agar	Anaerobic	50 mg/L pyrazosulfuron
24	Optimum substrate agar	Anaerobic	50 mg/L chloramphenicol
25	Optimum substrate agar	Anaerobic	50 mg/L penicillin
26	Pit muds	Anaerobic	–
27	Pit muds	Anaerobic	20 g/L glucose

### Strain Isolation

Serial dilutions of PM were prepared with sterilized water. Two hundred microliters of each dilution were directly inoculated on various culture media in an aerobic environment or anaerobic cycle system. The samples were cultured at 37°C for 40 days. Obligate anaerobic bacteria were cultured and isolated in a forma anaerobic system (Thermo Fisher Scientific). This anaerobic system was filled with the standard equilibrium gas N_2_:CO_2_:H_2_ = 85:10:5 (v/v). Single colonies were picked and isolated once every 2–15 days. At the beginning of isolation, the mono-clone was picked with a shorter time interval. With the growth of culture time, the mono-clone was picked with a longer time interval. The mono-clone was purified at least three times under the original culture conditions.

### Species Identification

Monoclonal genomic DNA was extracted with a TIANGEN Bacterial DNA kit (TIANGEN Biotech Beijing Co., Ltd., Beijing, China) according to the manufacturer’s instructions. Universal primers 27F (5′-AGAGTTTGATCCTGGCTCAG-3′) and 1492R (5′-TACGGCTACCTTGTTACGACTT-3′) were used to amplify the bacterial 16S rRNA gene sequence. The 25 μl PCR amplification reaction system contained 12.5 μl of 2 × Pfu PCR Master Mix (TIANGEN Biotech Beijing Co., Ltd., Beijing, China), 7.5 μl of ddH_2_O, 3 μl of DNA template, 1 μl of primer 27F, and 1 μl of primer 1492R as described in a previous study (Xu J. et al., 2019). The bacterial 16S rRNA gene was amplified according to the following procedure: 95°C for 5 min and 35 cycles at 95°C for 20 s, 55°C for 20 s, 72°C for 60 s, and 72°C for 5 min). The PCR product was sequenced by Sangon Biotech Co., Ltd. (Shanghai, China). Sequencing results were analyzed by the NCBI BLAST algorithm^4^ and EzTaxon-e BLAST^5^ (Yoon et al., 2017) for homologous sequence searches with type strains. If 16S rRNA is <98.65% similar to the closest type strain, the isolate could be a new species (Lagier et al., 2018). In addition, the similarity of 16S rRNA between species within the genus was also a reference for suspected new species. If the similarity of 16S rRNA is within the range, which the similarity has already been reported in the same genus.

### Phylogenetic Tree Construction

Sequences of the 16S rRNA of isolated strains were aligned using the CLUSTALX program (Thompson et al., 1997). A phylogenetic tree was constructed using neighbor-joining (Saitou and Nei, 1987) with MEGA version 6.0 software with 1,000 replicate bootstrap values (Tamura et al., 2013). Evolutionary distances were calculated using the Kimura two-parameter model (Kimura, 1980).

## Results

### Bacterial Diversity of the Pit Muds Using 16S rRNA Gene Sequencing

**FIGURE 1:**
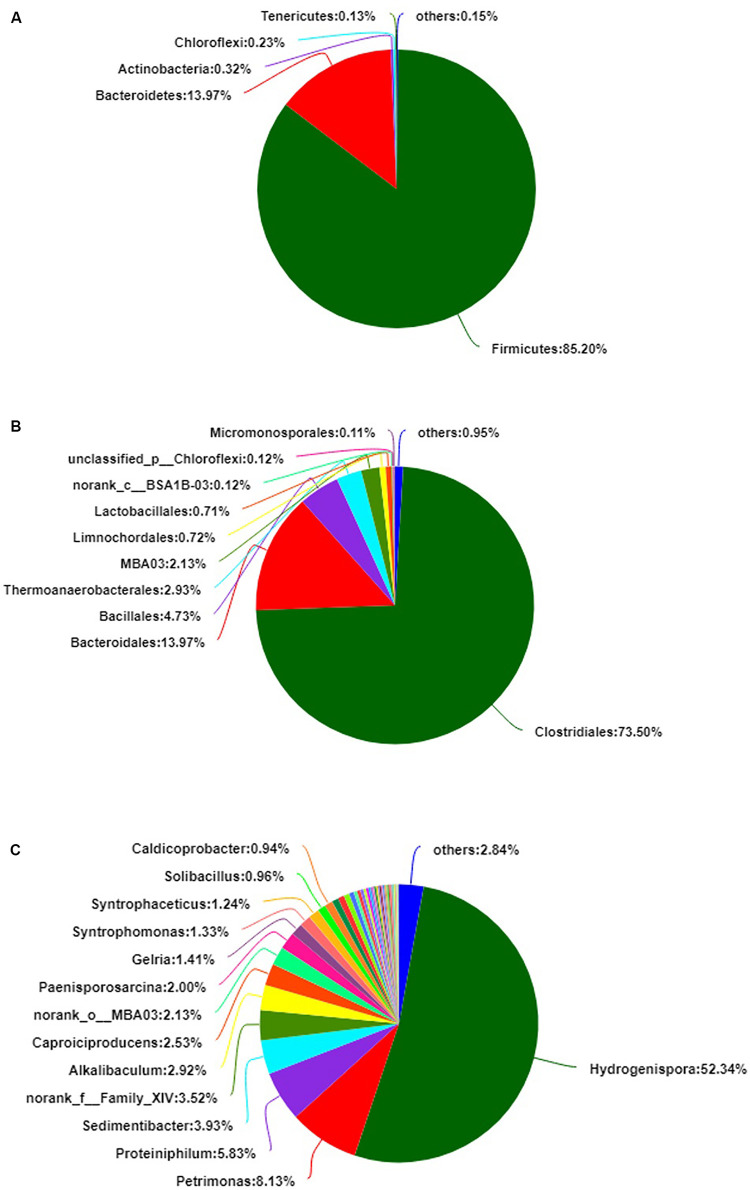
The relative abundance of bacteria in pit mud (PM). **(A)** Phylum level. **(B)** Order level. **(C)** Genus level.

A total of 141,228 reads were obtained by 16S rRNA gene sequencing and distributed to 467 OTUs. The alpha diversity, Shannon index, was 2.744 ± 0.165, and the Simpson index was 0.239 ± 0.037 (Supplementary Figure S1). The bacterial diversity of the PM microbiota consisted of nine phyla, 23 classes, 49 orders, 99 families, and 201 genera. The average of triplicate samples is shown in Figure 1. At the phylum level, Firmicutes and Bacteroidetes predominated (>99%) in the PM sample (Figure 1A). At the order level, Clostridiales, Bacteroidales, and Bacillales predominated (>92%) (Figure 1B). At the genus level, *Hydrogenispora*, *Petrimonas*, *Proteiniphilum*, and *Sedimentibacter* predominated (>70%) (Figure 1C). In the sample, anaerobic bacteria had the highest relative abundance (>80%), including *Hydrogenispora* (Liu Y. et al., 2014), *Petrimonas* (Grabowski et al., 2005), *Proteiniphilum* (Shuangya and Xiuzhu, 2005), *Sedimentibacter* (Imachi et al., 2016), *Alkalibaculum* (Allen et al., 2010), *Caproiciproducens* (Kim et al., 2015), *Gelria* (Plugge et al., 2002), and *Syntrophomonas* (Hatamoto et al., 2007). In addition, a relative abundance of approximately 10.10% of bacteria at the genus level was assigned to unclassified or no-rank. The community relative abundance is shown in Supplementary Tables S1–S5.

### Bacterial Diversity of Pit Muds Using Culturomics

**FIGURE 2:**
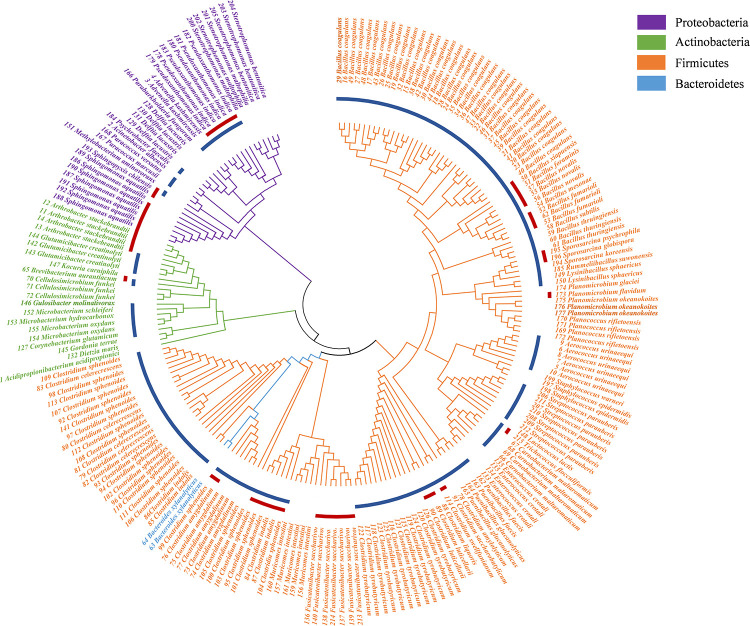
Neighbor-joining phylogenetic tree of isolated bacteria in pit mud (PM). The blue circle represents the genera detected by 16S rRNA gene sequencing; the red circle represents the potential new species.

In this study, a total of 27 culture conditions were tested: the common commercial medium, original environmental medium, and predicted medium with different gas compositions (Table 1). From the PM sample, a total of 215 colonies were obtained, and 66 bacterial species were observed. The bacteria belonged to 41 genera disseminated into four phyla: Firmicutes (74.41%), Actinobacteria (9.77%), Proteobacteria (14.88%), and Bacteroidetes (0.93%) (Figure 2, Table 2, and Supplementary Table S6). Among 41 genera obtained in pure culture in this study, 36 genera had never been isolated from PM in previous studies. *Bacillus* and *Clostridium* were dominant with 48 and 54 strains, respectively. However, only a single colony was obtained for *Acidipropionibacterium*, *Acinetobacter*, *Corynebacterium*, *Gordonia, Gulosibacter*, *Dietzia*, *Lactococcus*, *Methylobacterium*, *Rummeliibacillus*, *Psychrobacter*, *Kocuria*, and *Trichococcus*.

**TABLE 2:** Bacterial species isolated *via* culturomics in the pit muds (PMs).

**Bacterial species**	**Bacterial species**	**Bacterial species**	**Bacterial species**
*Acidipropionibacterium acidipropionici*^1^^∗^	*Cellulosimicrobium funkei*^1,11^^∗^	*Gordonia terrae*^4^^∗^	*Planomicrobium glaciei*^4^^∗^
*Acinetobacter albensis*^11^^∗^	*Clostridium amygdalinum*^18^	*Gulosibacter molinativorax*^1^^∗^	*Planomicrobium okeanokoites*^3,4^^∗^
*Advenella kashmirensis*^8,11^^∗^	*Clostridium amylolyticum*^17^	*Kocuria carniphila*^3^^∗^	*Pseudoxanthomonas indica*^4^^∗^
*Aerococcus urinaeequi*^10^^∗^	*Clostridium celerecrescens*^13,17^	*Lactococcus lactis subsp. Cremoris*^7^^∗^	*Psychrobacter faecalis*^4^^∗^
*Arthrobacter stackebrandtii*^2,3^^∗^	*Clostridium indolis*^17^	*Lysinibacillus sphaericus*^2,9^^∗^	*Rummeliibacillus suwonensis*^17^^∗^
*Bacillus coagulans*^13,21,23,27,14^	*Clostridium liquoris*^17^	*Methylobacterium aminovorans*^2^^∗^	*Sphingomonas aquatilis*^11^^∗^
*Bacillus xiapuensis*^4^	*Clostridium luticellarii*^17^	*Microbacterium schleiferi*^4^	*Sphingopyxis chilensis*^3^^∗^
*Bacillus foraminis*^4^	*Clostridium saccharobutylicum*^27^	*Microbacterium hydrocarbonoxydans*^4^	*Sporosarcina koreensis*^3^^∗^
*Bacillus fumarioli*^23^	*Clostridium sphenoides*^13,16,17,18,23^	*Microbacterium oxydans*^1^	*Sporosarcina psychrophila*^3^^∗^
*Bacillus mesonae*^4^	*Clostridium swellfunianum*^18^	*Muricomes intestine*^17^^∗^	*Sporosarcina globispora*^3^^∗^
*Bacillus novalis*^13,23^	*Clostridium tyrobutyricum*^12,13,14,17,27^	*Paenibacillus faecis*^18^	*Staphylococcus epidermidis*^1^^∗^
*Bacillus subtilis*^3^	*Corynebacterium glutamicum*^11^	*Paenibacillus glucanolyticus*^9,11^	*Staphylococcus warneri*^3^^∗^
*Bacillus thruingiensis*^4,18^	*Delftia lacustris*^2,4^^∗^	*Paraburkholderia fungorum*^11^	*Stenotrophomonas maltophilia*^8,11^^∗^
*Planomicrobium flavidum*^3^^∗^	*Dietzia maris*^11^^∗^	*Paracoccus marcusii*^10^	*Stenotrophomonas bentonitica*^2,3,4^^∗^
*Bacteroides xylanolyticus*^17,18^^∗^	*Enterococcus crotali*^1,3^^∗^	*Paracoccus versutus*^9^	*Streptococcus parauberis*^3,10^^∗^
*Brevibacterium aurantiacum*^4^^∗^	*Fusicatenibacter saccharivorans*^17,18^^∗^	*Planococcus rifietoensis*^3^	*Trichococcus flocculiformis*^3^^∗^
*Carnobacterium maltaromaticum*^7,11^^∗^	*Glutamicibacter creatinolyticus*^8^^∗^		

*Upper corner number is the culture condition No. ^∗^Indicates that these bacteria were isolated from PMs for the first time.*

### Comparing the Bacteria Found in the Pit Muds Using Culturomics and 16S rRNA Gene Sequencing

**FIGURE 3:**
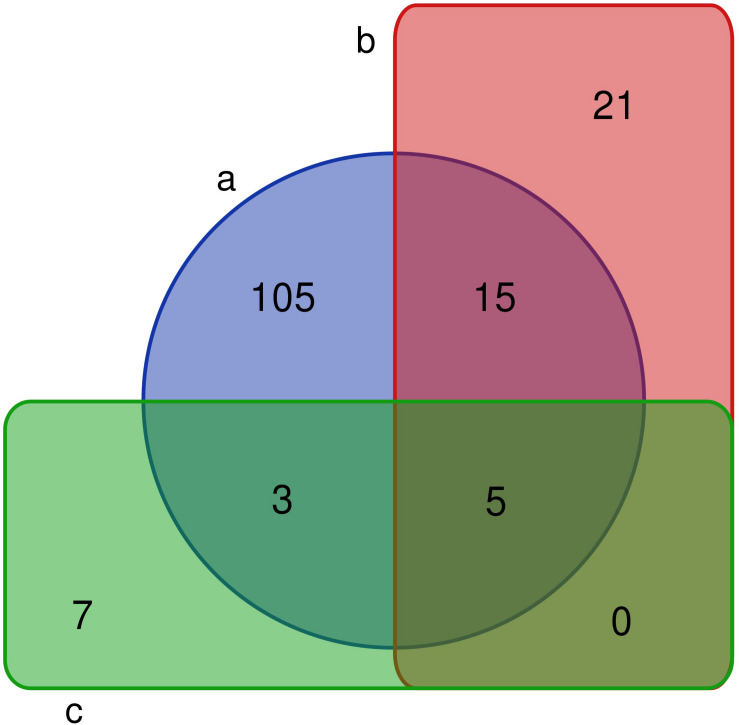
Venn diagram of bacterial genera in fermentation pit mud (PM). **(a)** The number of bacteria detected by 16S rRNA gene sequencing. **(b)** The number of fermentation PM culturable bacteria in this study. **(c)** The number of fermentation PM culturable bacteria in previous studies.

A comparative analysis showed that 4/8 bacterial phyla, 7/17 classes, 13/33 orders, 26/66 families, and 41/128 genera were observed in the PM using culturomics. At the phylum level, representatives of the main phyla (Firmicutes, Bacteroidetes, Actinobacteria, and Proteobacteria) identified by 16S amplicon sequencing were isolated by culturomics. Dominant bacterial classes and orders were isolated with relative abundances of 98.59 and 93.09%, respectively. However, bacterial families and genera were isolated with relative abundances of only 5.39 and 1.57%, respectively. Six families and 20 genera (*Acidipropionibacterium*, *Acinetobacter*, *Arthrobacter*, *Bacteroides*, *Delftia*, *Cellulosimicrobium*, *Dietzia*, *Enterococcus*, *Fusicatenibacter*, *Gordonia*, *Kocuria*, *Lactococcus*, *Methylobacterium*, *Muricomes*, *Paraburkholderia*, *Planococcus*, *Planomicrobium*, *Sphingomonas*, *Staphylococcus*, and *Stenotrophomonas*) were isolated but not detected by 16S rRNA gene sequencing (Figure 3).

In addition to previously known bacteria, 19 potential new species (47 strains) were isolated from the Baijiu PM sample analyzed *via* culturomics, including one potential new genus and 18 potential new species belonging to *Bacillus*, *Clostridium*, *Sporosarcina*, *Bacteroides*, and *Trichococcus*. The potential new species were obtained from potato dextrose agar (PDA), tryptic soy agar (TSA), R2A, DSMZ-500, or optimum substrate agar. Culture condition No. 23 was suitable for the discovery and isolation of novel *Bacillus* species, culture condition No. 17 was suitable for the discovery and isolation of novel *Clostridium* species, and culture condition No. 3 was suitable for the discovery and isolation of aerobic microorganisms (Table 3).

**TABLE 3:** Potentially new bacterial species in the pits muds (PMs).

**Strain No.**	**Culture conditions No.**	**Closest type species**	**Similarity**	**Reference similarity within the genus**
NG9	2	*Arthrobacter stackebrandtii*	99.00	93.5–99.4% (Chang et al., 2007; Yassin et al., 2011; SantaCruz-Calvo et al., 2013)
NG3	2	*Arthrobacter stackebrandtii*	99.07	
NF33	3	*Arthrobacter stackebrandtii*	99.07	
NF46	3	*Arthrobacter stackebrandtii*	99.07	
NC32A	4	*Bacillus xiapuensis*	97.54	
NC35B	4	*Bacillus foraminis*	98.43	
KB18	23	*Bacillus fumarioli*	98.15	
KB10	23	*Bacillus fumarioli*	97.58	
KB11	23	*Bacillus fumarioli*	97.93	
F11B	13	*Bacillus novalis*	97.18	
KB26	23	*Bacillus novalis*	97.26	
KB27B	23	*Bacillus novalis*	97.26	
NC35A	4	*Brevibacterium aurantiacum*	98.50	
JB5	18	*Clostridium amygdalinum*	97.73	
JA81	17	*Clostridium indolis*	95.43	
JA30	17	*Clostridium indolis*	95.85	
JA3	17	*Clostridium luticellarii*	97.05	
JA44	17	*Clostridium sphenoides*	95.81	
JA75	17	*Clostridium sphenoides*	95.73	
JA87	17	*Clostridium sphenoides*	95.80	
JA58	17	*Clostridium sphenoides*	95.91	
JA33	17	*Clostridium sphenoides*	97.61	
JB67	18	*Clostridium swellfunianum*	95.66	
JA74	17	*Fusicatenibacter saccharivorans*	92.49	
JA46	17	*Fusicatenibacter saccharivorans*	92.68	
JA37	17	*Fusicatenibacter saccharivorans*	93.00	
JA46B	17	*Fusicatenibacter saccharivorans*	93.00	
JA65	17	*Fusicatenibacter saccharivorans*	93.00	
JA70	17	*Fusicatenibacter saccharivorans*	93.00	
JA73	17	*Fusicatenibacter saccharivorans*	93.00	
NF36	3	*Planomicrobium flavidum*	98.38	
NC11	4	*Pseudoxanthomonas indica*	98.98	96.5–99.00% (Zhang et al., 2014)
NC12	4	*Pseudoxanthomonas indica*	99.11	
NC10	4	*Pseudoxanthomonas indica*	99.19	
NC19	4	*Pseudoxanthomonas indica*	99.19	
NC23	4	*Pseudoxanthomonas indica*	99.19	
NC24	4	*Pseudoxanthomonas indica*	99.19	
I35	11	*Sphingomonas aquatilis*	97.86	
I44	11	*Sphingomonas aquatilis*	97.86	
I16A	11	*Sphingomonas aquatilis*	97.94	
I45A	11	*Sphingomonas aquatilis*	98.00	
I42	11	*Sphingomonas aquatilis*	98.06	
I40	11	*Sphingomonas aquatilis*	98.27	
NF25	3	*Sphingopyxis chilensis*	99.11	<99.15% (Oelschlaegel et al., 2015)
NF7	3	*Sporosarcina globispora*	98.12	93.00–99.2% (Reddy et al., 2003; Tominaga et al., 2009)
NF26	3	*Sporosarcina psychrophila*	98.90	
NF24	3	*Trichococcus flocculiformis*	99.86	99.7–99.9% (Parshina et al., 2019)

### The Effect of Culture Conditions on Bacterial Culture

According to the results of 16S rRNA gene sequencing, aerobic bacteria occupied a low relative abundance (<20%). Four kinds of common commercial media and two original environmental media were used to isolate aerobic bacteria. Five kinds of common commercial media were set in microanaerobic environments. However, four common commercial mediums, 10 predicted mediums, and two original environmental mediums were used for isolating anaerobic bacteria. *Hydrogenispora*, which is an anaerobic, spore-forming, ethanol-hydrogen-coproducing bacterium (Liu Y. et al., 2014), had the highest relative abundance. *Hydrogenispora ethanolica* ferments glucose, maltose, arabinose, fructose, xylose, ribose, sucrose, galactose, mannose, pectin, starch, glycerol, tryptone, and yeast extract in pure culture. It is resistant to ampicillin, pyrazosulfuron, chloramphenicol, and penicillin (Liu Y. et al., 2014). According to the 16S rRNA of *Hydrogenispora ethanolica* (unique type strain in *Hydrogenispora*), DSMZ_Medium500 *Clostridium* medium was selected. Finally, DSMZ_Medium500 and suitable fermentation substrate medium with ampicillin, pyrazosulfuron, chloramphenicol, or penicillin were used.

More plentiful bacteria were obtained in aerobic environments. Sixty-two strains of 35 species, belonging to 23 genera, 18 families, 10 orders, 5 classes, and 3 phyla, were isolated. TSA and R2A have the best isolation ability between different genera in aerobic environments. In the microanaerobic environment, 38 strains of 20 species, belonging to 16 genera, 15 families, 9 orders, 5 classes, and 3 phyla, were isolated, and Gaoshi No. 1 culture medium had the best ability to isolate genera. In the anaerobic environment, *Clostridium* and *Bacillus* were largely isolated, including 98 strains and at least 18 species. In addition, *Bacteroides*, *Muricomes*, *Paenibacillus*, and *Rummeliibacillus* were also isolated from anaerobic environments. More single colonies were obtained on DSMZ-500 medium supplemented with 50 mg/l ampicillin. Among the 27 different culture conditions tested, the most effective condition was an anaerobic atmosphere. Different dominant microbial genera were separated under different gas conditions. The use of antibiotics also resulted in the different growth of bacteria with antibiotic resistance.

On the other hand, no species were isolated under the No. 5, No. 6, No. 15, No. 19, No. 20, No. 22, No. 24, No. 25, and No. 26 culture conditions. These culture conditions are ineffective for isolating bacteria from Baijiu PM. The No. 5, No. 6, No. 26, and No. 27 culture conditions indicated that PM is ineffective for isolating bacteria because of the oligotrophic environment of PM. The addition of glucose helped the growth of bacteria. Gaoshi No.1 medium can only be used in aerobic environments. The presence of chloramphenicol and penicillin can inhibit the growth of culturable microorganisms in PM and is not suitable for the isolation and culture of Baijiu fermentation bacteria. *Clostridium* and *Bacillus* are not resistant to these two antibiotics.

## Discussion

The purpose of this study was to evaluate the bacterial diversity of PM by 16S amplicon sequencing and culturomic approaches. At the class level, Limnochordia and Mollicutes, whose average relative abundance was >0.1%, were not isolated. The orders Thermoanaerobacterales, Limnochordales, and Micromonosporales, with average relative abundances >0.1%, were not isolated either. However, most bacteria with a high relative abundance were isolated at the class and order levels. Culturomics and 16S rRNA gene sequencing were verified mutually. However, at the family level, Heliobacteriaceae (52.68%) and Porphyromonadaceae (13.72%) were not isolated, and at the genus level, *Hydrogenispora* (52.68%), *Petrimonas* (7.92%), *Proteiniphilum* (5.80%), *Sedimentibacter* (3.93%), *Alkalibaculum* (2.94%), *Caproiciproducens* (2.51%), *Paenisporosarcina* (1.97%), and *Gelria* (1.42%) were not isolated. The results of culturomics and 16S rRNA gene sequencing showed significant differences at the family and genus levels. Indeed, a previous study found that only 15% of species were identified using either approach (Lagier et al., 2012). The difference between these two omics methods reveals that most microbial species in the biosphere resist cultivation in the laboratory even though they provide the chemical components of the natural environment. Many studies found that microbial species that would be “culturable” may fail to grow because of their growth state of dormancy (Connon and Giovannoni, 2002); these microorganisms are referred to as viable but non-culturable (VBNC) (Xu et al., 1982). According to 16S rRNA gene sequencing, PM may be a seed bank, but cells might be dormant because of the lack of a major nutrient or carbon source even though they naturally occur in PM. As a result, dormant microbes cannot easily be captured even through providing their natural environment. Therefore, enriched culturing and induction treatment may be future research directions to isolate VBNC bacteria in PM (Mu et al., 2018).

In the PM in this study, approximately 10.10% of bacteria were assigned to unclassed or no-rank at the genus level from 16S rRNA gene sequencing data. OTUs generated by 16S rRNA gene sequencing consist of clusters of DNA sequences and are used for classifying groups of microorganisms that are closely related (Caporaso et al., 2010). However, classification at the species level and the presence of spurious OTUs that overestimate the true diversity were still a challenge, and integrating OTUs in 16S rRNA gene sequencing is important for deciphering the microbiota diversity. However, sequencing of the 16S rRNA hypervariable regions is the gold standard for taxonomic assignment (Lagier et al., 2018). A recent study enabled unidentified species to be classified by integrating 16S rRNA gene sequencing and culturomics (Lagier et al., 2016). Culturomics could reduce the number of these unclassed or no-rank OTUs by increasing the number of pure cultured microorganism species. Therefore, this study is important to reduce the number of these unclassed or no-rank genera in PM.

In previous studies, 15 genera were isolated from PM by traditional methods, including *Clostridium*, *Paenibacillus* (Chen et al., 2015), *Bacillus* (Huang et al., 2014; Ma et al., 2016), *Sporolactobacillus* (Yue et al., 2007), *Lysobacter* (Zhang et al., 2017), *Mycobacterium*, *Pseudomonas*, *Microbacterium*, *Corynebacterium*, *Flavobacterium* (Yue et al., 2007), *Mierococcus*, *Staphylococcus*, *Burkholderia* (Huang et al., 2014), *Brevibacillus*, and *Aneurinibacillus* (Huang et al., 2014). In this study, 41 genera were isolated and obtained from pure culture, and 34 genera were never isolated from PM in previous studies. The results revealed that at least 32% of the bacterial genera in the PM in this study were culturable. Typically, only 0.1–10% of microorganisms are culturable. With culturomics, the diversity spectrum of culturable bacteria in the PM increased 273.33% at the genus level. According to the phylogenetic tree, Firmicutes was the phylum with the most strains. However, many strains were identified as potential new species in Firmicutes. Therefore, the culture of culturable bacteria in PM is still a long-term effort requiring continuous exploration.

Microbial diversity in different PM has been widely revealed in recent years. Liu et al. (2020) found that *Clostridium* and *Bacillus* were dominant bacterial genera in PM with high relative abundance during Chinese Baijiu production. However, there are significant differences in microbial community structure in different PM samples. The structure of the microbial community in PM is related to the age of the cellar. A previous study showed that the diversity of the bacterial community in PM samples was relatively stable in cellars aged 20–30 years. In cellars of 10–30 years, the relative abundance of *Ruminococcaceae* and *Clostridium* in PM increased, while that of *Petrimonas* and Firmicutes decreased (Tan et al., 2020). In addition, the decrease in the relative abundance of *Lactobacillus* and the rapid increase in *Clostridium* and *Aminobacterium* in unique, absolute dominant bacteria indicated that the PM had become a high-quality pit that tended to mature (Wang et al., 2019). In general, *Clostridium* was the dominant bacteria in PM in these studies. This is consistent with the culturomic results of this study. However, this result is different from the 16S rRNA gene sequencing results, which indicated that *Hydrogenispora* is the dominant bacteria in this study. *Hydrogenispora* and *Clostridium* are both Clostridiales. Therefore, they may have similar functions in fermenting Baijiu.

Among these pure-culture microorganisms, *Clostridium* is a common anaerobe that is usually found within the Baijiu PM. *Clostridium* is one of the most important bacteria for the formation of the main flavor compounds of Baijiu (Zou et al., 2018). *Clostridium* has a broad spectrum of substrate utilization, and its main products are organic acids, ethanol, and hydrogen. The organic acids can be esterified to ester. Hydrogen can support the stability of the microbial community in PM. *Clostridium* can synthesize short-chain fatty acids, which are the precursors of the main flavor compounds of Baijiu (Zou et al., 2018). In addition, Clostridia was the major group possessing the butyrate kinase (buk) pathway and dominated the butyryl-CoA:acetate CoA-transferase (but) pathway. Clostridia in the PM mainly synthesize butyric acid, which is the key aroma contributor of strong-flavor Baijiu, through the buk pathway (Chai et al., 2019). At present, a total of 26 *Clostridium* species have been isolated and identified in the PM of different Baijiu distilleries (Zou et al., 2018; Xu P. et al., 2019). In this study, *C*. *sphenoides*, *C*. *amygdalinum*, and *C*. *saccharobutylicum* and five potentially novel *Clostridium* species were isolated from PM for the first time. This study extends the species diversity of *Clostridium* in PM in a culture-dependent manner. Further research on the function and metabolism of *Clostridium* in PM is important.

*Bacillus* is one of the most common bacterial genera, with approximately 270 species isolated and cultured due to spore resistance. Eight species of *Bacillus* were isolated from the PM in this study, and *Bacillus coagulans* was the most isolated species among *Bacillus* in the PM. *B. coagulans* was only isolated from anaerobic atmosphere, but it is facultative anaerobic bacterium. *B. coagulans* was first isolated from spoiled milk (Kristjansson, 1991). *B. coagulans* is resistant to high temperatures and has probiotic activity. A large number of studies have been carried out on the low-cost microbial production of industrially valuable products that have been used in food production (Konuray and Erginkaya, 2018). *B. coagulans* can metabolize and produce lactic acid, β-galactosidase, α-amylase (Babu and Satyanarayana, 1995; Keating et al., 1998), lipase (Tang and Xia, 2005; Gowthami et al., 2015), and xylanase (Heck et al., 2005; Chauhan et al., 2006). Among these metabolites, lactic acid is an important product based on its high yield (Zhou et al., 2016). β-Galactosidase is an important enzyme in the food industry with many applications. Amylases are important hydrolase enzymes that can be produced from plants and microorganisms. Lipase is widely used in the food industry as well as in the pharmaceutical, textile, and cosmetic industries (Satyendra et al., 2005; Kanwar et al., 2006). Xylanase is used in the clarification process of fruit juice and wine. In addition, *B. coagulans* has proteolytic activity. *B. coagulans*, which was isolated from the PM in this study, may have similar functions during the Baijiu-making process. However, this requires further exploration, and pure culture of *B. coagulans* isolated from PM is the basis of future research. The role of supporting evidence through comparative genomics (sequencing the new isolates) and experiments on new species isolated for the discovery of new metabolic traits and functions to understand the Baijiu properties should be interesting.

The combination of 16S rRNA gene sequencing and culturomics more fully revealed the bacterial diversity of PM. The two omics methods confirm and complement each other. 16S rRNA gene sequencing directs microbial culturomics studies. Culturomics can be used to obtain pure cultures of microorganisms and help determine unknown OTUs. It is the foundation for the subsequent exploration of microbial function. This study is the first to apply culturomics to Baijiu-making microorganisms in the food industry. It enriched the microbial pure culture library of PM and allowed a number of potential new species to be obtained. It is of great significance to reveal the mechanism and microbial function of Baijiu brewing in the future.

## Data Availability Statement

The sequencing data has been submitted to NCBI with accession number SRR11624724.

## Author Contributions

QR and JX contributed to the methodology. LS contributed to writing the original draft preparation. XX and HG contributed to the experiment. ZS contributed to the writing, reviewing, and editing. XL and ZL contributed to the supervision. All authors contributed to the article and approved the submitted version.

## Conflict of Interest

The authors declare that the research was conducted in the absence of any commercial or financial relationships that could be construed as a potential conflict of interest.

## Footnotes

1 http://drive5.com/uparse/

2 http://mothur.org/wiki/miseq_sop/#alpha-diversity-1

3 http://komodo.modelseed.org

4 https://blast.ncbi.nlm.nih.gov

5 https://www.ezbiocloud.net

## Abbreviations

KOMODO: Known Media Database
OTUs: operational taxonomic units
PDA: potato dextrose agar
PM: pit mud
TSA: tryptic soy agar
VBNC: viable but non-culturable.
