# Supplementary material for: Culturing Bacteria From Fermentation Pit Muds of Baijiu With Culturomics and Amplicon-Based Metagenomic Approaches
Source: Front Microbiol. 2020 Jun 23;11:1223. doi: 10.3389/fmicb.2020.01223 (PMC7344326; doi:10.3389/fmicb.2020.01223)
Supplement: Supplementary file 1 [file Data_Sheet_1.docx]

Culturing bacteria from fermentation pit muds of baijiu with culturomics and amplicon-based metagenomic approaches

Jialiang Xu^1,#^, Leping Sun^1,#^, Xuan Xing^1^, Zhanbin Sun^1^, Haoyue Gu^1^, Xin Lu^2^, Zhenpeng Li^2^, Qing Ren^1,*^

**Author affiliations:**

1. Beijing Technology and Business University, Beijing 100048, China.

2. State Key Laboratory for Infectious Disease Prevention and Control, National Institute for Communicable Disease Control and Prevention, Chinese Center for Disease Control and Prevention, Beijing 102206, China.

Corresponding author: Qing Ren ([renqing@th.btbu.edu.cn](mailto:renqing@th.btbu.edu.cn))

Other author: Jialiang Xu ([xujialiang@btbu.edu.cn](mailto:xujialiang@btbu.edu.cn))

Leping Sun ([liposun@163.com](mailto:liposun@163.com))

Xuan Xing ([1053808937@qq.com](mailto:1053808937@qq.com))

Zhanbin Sun ([twins5616@126.com](mailto:twins5616@126.com))

Haoyue Gu ([2429431338@qq.com](mailto:2429431338@qq.com))

Xin Lu ([luxin@icdc.cn](mailto:luxin@icdc.cn))

Zhenpeng Li ([lizhenpeng@icdc.cn](mailto:lizhenpeng@icdc.cn))

^#^These authors contributed equally to this work.


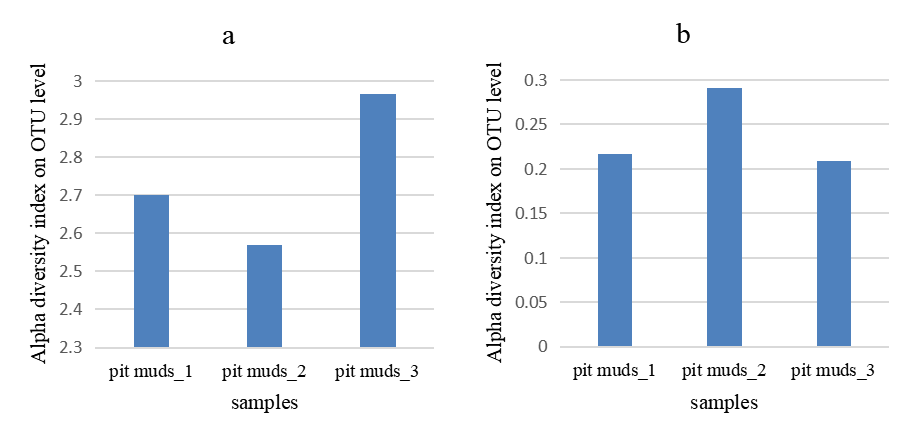


Fig. S1. Alpha diversity index on OTU level. (a. means Shannon index. b. means Simpson index)


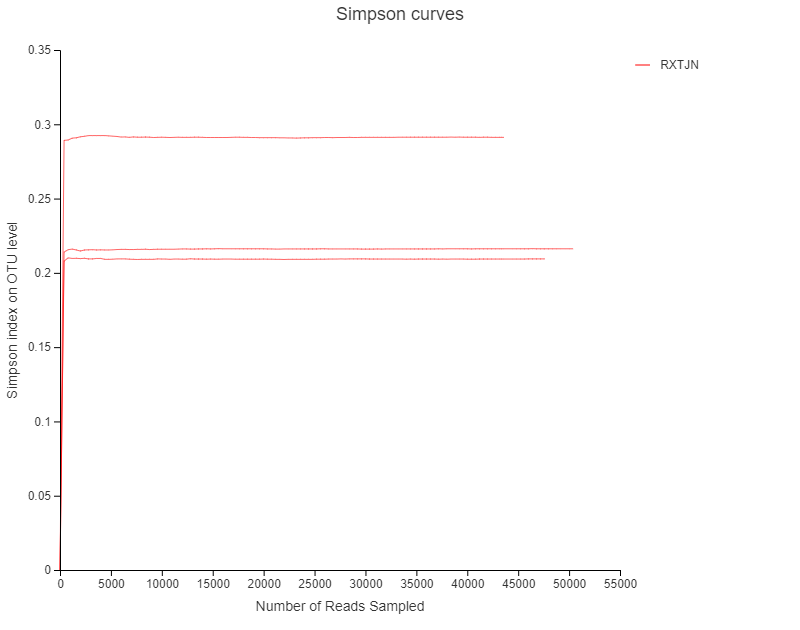


Fig. S2. The rarefaction curves of samples with Simpson index on OTU level.

Table S1. The relative abundance of bacteria in pit muds on phylum level

| OTU ID | 1 | 2 | 3 | Average |
| --- | --- | --- | --- | --- |
| Firmicutes | 0.78699 | 0.88958 | 0.88631 | 0.854293314 |
| Bacteroidetes | 0.207513 | 0.096931 | 0.107767 | 0.137403704 |
| Actinobacteria | 0.00125 | 0.006302 | 0.002097 | 0.003216452 |
| Proteobacteria | 0.000397 | 0.000717 | 0.00053 | 0.000547856 |
| Chloroflexi | 0.001707 | 0.003731 | 0.001268 | 0.002234917 |
| unclassified_k_norank | 0.001052 | 0.000801 | 0.000807 | 0.000886434 |
| Tenericutes | 0.001012 | 0.001792 | 0.001152 | 0.001318638 |
| Synergistetes | 7.94E-05 | 8.43E-05 | 6.91E-05 | 7.76081E-05 |
| Gemmatimonadetes | 0 | 6.32E-05 | 0 | 2.10766E-05 |

Table S2. The relative abundance of bacteria in pit muds on class level

| OTU ID | 1 | 2 | 3 | Average |
| --- | --- | --- | --- | --- |
| Clostridia | 0.711998 | 0.82635 | 0.833994008 | 0.790780633 |
| Bacteroidia | 0.207493 | 0.096868 | 0.10774372 | 0.13736833 |
| Bacilli | 0.066062 | 0.057539 | 0.039248675 | 0.054283282 |
| Limnochordia | 0.007323 | 0.003709 | 0.010901129 | 0.007311067 |
| Actinobacteria | 0.00125 | 0.006302 | 0.002097257 | 0.003216452 |
| Mollicutes | 0.001012 | 0.001792 | 0.001152339 | 0.001318638 |
| Anaerolineae | 0.000933 | 0.000443 | 0.000207421 | 0.000527572 |
| Gammaproteobacteria | 0.000198 | 0.000379 | 0.000184374 | 0.000254066 |
| Alphaproteobacteria | 0.000159 | 0.00019 | 0.000161327 | 0.000169924 |
| Synergistia | 7.94E-05 | 8.43E-05 | 6.91404E-05 | 7.76081E-05 |
| Betaproteobacteria | 0 | 6.32E-05 | 0.000138281 | 6.71702E-05 |
| Deltaproteobacteria | 3.97E-05 | 8.43E-05 | 4.60936E-05 | 5.66963E-05 |
| Thermomicrobia | 1.98E-05 | 2.11E-05 | 2.30468E-05 | 2.13226E-05 |
| Gemmatimonadetes | 0 | 6.32E-05 | 0 | 2.10766E-05 |
| Flavobacteriia | 0 | 4.22E-05 | 0 | 1.40511E-05 |
| Cytophagia | 1.98E-05 | 2.11E-05 | 0 | 1.36403E-05 |
| Sphingobacteriia | 0 | 0 | 2.30468E-05 | 7.68226E-06 |
| unclassified | 0.002123 | 0.003878 | 0.002258585 | 0.002753344 |
| Others | 0.00129 | 0.002171 | 0.001751556 | 0.001737444 |

Table S3. The relative abundance of bacteria in pit muds on order level

| OTU ID | 1 | 2 | 3 | Average |
| --- | --- | --- | --- | --- |
| Clostridiales | 0.641471 | 0.793049 | 0.780272 | 0.73826392 |
| Bacteroidales | 0.207493 | 0.096868 | 0.107744 | 0.13736833 |
| Bacillales | 0.057509 | 0.045842 | 0.036921 | 0.046757222 |
| Thermoanaerobacterales | 0.031771 | 0.02076 | 0.035838 | 0.02945637 |
| Others | 0.038796 | 0.013995 | 0.017331 | 0.02337396 |
| Limnochordales | 0.007323 | 0.003709 | 0.010901 | 0.007311067 |
| Lactobacillales | 0.008414 | 0.01037 | 0.002028 | 0.006937278 |
| unclassified | 0.002401 | 0.004553 | 0.003572 | 0.003508657 |
| norank | 0.00129 | 0.002213 | 0.001752 | 0.001751495 |
| Micromonosporales | 0.000337 | 0.002276 | 0.00083 | 0.00114777 |
| Haloplasmatales | 0.000853 | 0.001012 | 0.000714 | 0.000859812 |
| Streptosporangiales | 0.000298 | 0.001644 | 0.000461 | 0.000800859 |
| Corynebacteriales | 0.000337 | 0.001454 | 0.000254 | 0.000681718 |
| Anaerolineales | 0.000933 | 0.000443 | 0.000207 | 0.000527572 |
| Micrococcales | 0.000179 | 0.000653 | 0.000461 | 0.00043097 |
| Pseudomonadales | 0.000139 | 0.000232 | 0.000138 | 0.000169678 |
| Rhizobiales | 9.92E-05 | 0.00019 | 0.000138 | 0.000142397 |
| Synergistales | 7.94E-05 | 8.43E-05 | 6.91E-05 | 7.76081E-05 |
| Burkholderiales | 0 | 6.32E-05 | 0.000138 | 6.71702E-05 |
| Frankiales | 3.97E-05 | 0.000105 | 4.61E-05 | 6.37218E-05 |
| Myxococcales | 3.97E-05 | 8.43E-05 | 4.61E-05 | 5.66963E-05 |
| Coriobacteriales | 3.97E-05 | 4.22E-05 | 4.61E-05 | 4.26452E-05 |
| Methylococcales | 3.97E-05 | 2.11E-05 | 4.61E-05 | 3.56197E-05 |
| Xanthomonadales | 0 | 0.000105 | 0 | 3.51277E-05 |
| Acidimicrobiales | 0 | 4.22E-05 | 0 | 1.40511E-05 |
| Flavobacteriales | 0 | 4.22E-05 | 0 | 1.40511E-05 |
| Pseudonocardiales | 0 | 4.22E-05 | 0 | 1.40511E-05 |
| Propionibacteriales | 1.98E-05 | 2.11E-05 | 0 | 1.36403E-05 |
| Aeromonadales | 1.98E-05 | 2.11E-05 | 0 | 1.36403E-05 |
| Cytophagales | 1.98E-05 | 2.11E-05 | 0 | 1.36403E-05 |
| Sphingomonadales | 3.97E-05 | 0 | 0 | 1.32296E-05 |
| Rhodospirillales | 0 | 0 | 2.3E-05 | 7.68226E-06 |
| Sphingobacteriales | 0 | 0 | 2.3E-05 | 7.68226E-06 |
| Gemmatimonadales | 0 | 2.11E-05 | 0 | 7.02553E-06 |
| Streptomycetales | 0 | 2.11E-05 | 0 | 7.02553E-06 |
| Rhodobacterales | 1.98E-05 | 0 | 0 | 6.61481E-06 |

Table S4. The relative abundance of bacteria in pit muds on family level

| OTU ID | 1 | 2 | 3 | Average |
| --- | --- | --- | --- | --- |
| Heliobacteriaceae | 0.473111 | 0.49981 | 0.60749 | 0.526803776 |
| Porphyromonadaceae | 0.207493 | 0.096383 | 0.107698 | 0.137191379 |
| Others | 0.087137 | 0.092948 | 0.078543 | 0.086209354 |
| Planococcaceae | 0.048162 | 0.037053 | 0.030652 | 0.038622427 |
| Ruminococcaceae | 0.024171 | 0.064284 | 0.018898 | 0.035784158 |
| norank | 0.04721 | 0.020107 | 0.029592 | 0.032303005 |
| Eubacteriaceae | 0.014387 | 0.056738 | 0.023623 | 0.031582782 |
| Thermoanaerobacteraceae | 0.028616 | 0.018821 | 0.03298 | 0.026805667 |
| Syntrophomonadaceae | 0.021154 | 0.026915 | 0.019797 | 0.022622049 |
| Caldicoprobacteraceae | 0.005715 | 0.015196 | 0.00749 | 0.009467207 |
| Clostridiaceae | 0.003294 | 0.010939 | 0.010071 | 0.008101457 |
| unclassified | 0.008374 | 0.007166 | 0.006983 | 0.007507854 |
| Carnobacteriaceae | 0.008136 | 0.009758 | 0.001198 | 0.006364369 |
| Lachnospiraceae | 0.003135 | 0.007946 | 0.004678 | 0.005253264 |
| Clostridiales | 0.002977 | 0.006934 | 0.003803 | 0.004571194 |
| Peptococcaceae | 0.00508 | 0.004173 | 0.004287 | 0.004513346 |
| Christensenellaceae | 0.002659 | 0.005164 | 0.002166 | 0.003329772 |
| Gracilibacteraceae | 0.001766 | 0.003815 | 0.002212 | 0.002597836 |
| Thermoactinomycetaceae | 0.001131 | 0.002361 | 0.001314 | 0.001601792 |
| Bacillaceae | 0.001052 | 0.001686 | 0.000714 | 0.001150777 |
| Micromonosporaceae | 0.000337 | 0.002276 | 0.00083 | 0.00114777 |
| Haloplasmataceae | 0.000853 | 0.001012 | 0.000714 | 0.000859812 |
| Streptosporangiaceae | 0.000298 | 0.001602 | 0.000461 | 0.000786808 |
| Paenibacillaceae | 0.000278 | 0.00137 | 0.000668 | 0.000772052 |
| Limnochordaceae | 0.000913 | 0.000653 | 0.000507 | 0.000691082 |
| Corynebacteriaceae | 0.000337 | 0.001433 | 0.000254 | 0.000674693 |
| Anaerolineaceae | 0.000933 | 0.000443 | 0.000207 | 0.000527572 |
| Lactobacillaceae | 9.92E-05 | 0.00019 | 0.000645 | 0.000311407 |
| Microbacteriaceae | 5.95E-05 | 0.000379 | 0.000161 | 0.00020008 |
| Aerococcaceae | 0.000179 | 0.000232 | 0.000161 | 0.00019059 |
| Staphylococcaceae | 0.000278 | 0.000148 | 0.000138 | 0.00018788 |
| Marinilabiaceae | 0 | 0.000485 | 4.61E-05 | 0.000176952 |
| Micrococcaceae | 5.95E-05 | 0.000126 | 0.0003 | 0.000161867 |
| Moraxellaceae | 0.000139 | 8.43E-05 | 0.000115 | 0.000112817 |
| Synergistaceae | 7.94E-05 | 8.43E-05 | 6.91E-05 | 7.76081E-05 |
| Streptococcaceae | 0 | 0.00019 | 2.3E-05 | 7.0912E-05 |
| Pseudomonadaceae | 0 | 0.000148 | 2.3E-05 | 5.6861E-05 |
| Hyphomicrobiaceae | 5.95E-05 | 6.32E-05 | 2.3E-05 | 4.86033E-05 |
| Phyllobacteriaceae | 1.98E-05 | 4.22E-05 | 6.91E-05 | 4.37127E-05 |
| Coriobacteriaceae | 3.97E-05 | 4.22E-05 | 4.61E-05 | 4.26452E-05 |
| Promicromonosporaceae | 0 | 0.000126 | 0 | 4.21532E-05 |
| Alcaligenaceae | 0 | 2.11E-05 | 9.22E-05 | 3.77546E-05 |
| Methylococcaceae | 3.97E-05 | 2.11E-05 | 4.61E-05 | 3.56197E-05 |
| Peptostreptococcaceae | 1.98E-05 | 6.32E-05 | 2.3E-05 | 3.53737E-05 |
| Xanthomonadaceae | 0 | 0.000105 | 0 | 3.51277E-05 |
| Burkholderiaceae | 0 | 4.22E-05 | 4.61E-05 | 2.94156E-05 |
| Methylocystaceae | 0 | 6.32E-05 | 2.3E-05 | 2.87589E-05 |
| Nannocystaceae | 1.98E-05 | 2.11E-05 | 2.3E-05 | 2.13226E-05 |
| Phaselicystidaceae | 1.98E-05 | 0 | 2.3E-05 | 1.42971E-05 |
| Flavobacteriaceae | 0 | 4.22E-05 | 0 | 1.40511E-05 |
| Polyangiaceae | 0 | 4.22E-05 | 0 | 1.40511E-05 |
| Pseudonocardiaceae | 0 | 4.22E-05 | 0 | 1.40511E-05 |
| Thermomonosporaceae | 0 | 4.22E-05 | 0 | 1.40511E-05 |
| Propionibacteriaceae | 1.98E-05 | 2.11E-05 | 0 | 1.36403E-05 |
| Aeromonadaceae | 1.98E-05 | 2.11E-05 | 0 | 1.36403E-05 |
| Rhizobiaceae | 1.98E-05 | 2.11E-05 | 0 | 1.36403E-05 |
| Brevibacteriaceae | 3.97E-05 | 0 | 0 | 1.32296E-05 |
| Bradyrhizobiaceae | 0 | 0 | 2.3E-05 | 7.68226E-06 |
| Rhodospirillales_Incertae_Sedis | 0 | 0 | 2.3E-05 | 7.68226E-06 |
| Sphingobacteriaceae | 0 | 0 | 2.3E-05 | 7.68226E-06 |
| Cyclobacteriaceae | 0 | 2.11E-05 | 0 | 7.02553E-06 |
| Gemmatimonadaceae | 0 | 2.11E-05 | 0 | 7.02553E-06 |
| Iamiaceae | 0 | 2.11E-05 | 0 | 7.02553E-06 |
| Nocardiaceae | 0 | 2.11E-05 | 0 | 7.02553E-06 |
| Streptomycetaceae | 0 | 2.11E-05 | 0 | 7.02553E-06 |
| Rhodobacteraceae | 1.98E-05 | 0 | 0 | 6.61481E-06 |
| Sphingomonadaceae | 1.98E-05 | 0 | 0 | 6.61481E-06 |
| Cytophagaceae | 1.98E-05 | 0 | 0 | 6.61481E-06 |
| Erythrobacteraceae | 1.98E-05 | 0 | 0 | 6.61481E-06 |

Table S5. The relative abundance of bacteria in pit muds on genus level

| OTU ID | 1 | 2 | 3 | Average |
| --- | --- | --- | --- | --- |
| *Hydrogenispora* | 0.473110811 | 0.499810311 | 0.607490205 | 0.526803776 |
| *norank* | 0.107596444 | 0.065695738 | 0.075455174 | 0.082915785 |
| *Petrimonas* | 0.134207811 | 0.055157442 | 0.048283015 | 0.079216089 |
| *Proteiniphilum* | 0.073285442 | 0.041225815 | 0.059414612 | 0.057975289 |
| *Sedimentibacter* | 0.028933164 | 0.055009906 | 0.034063148 | 0.039335406 |
| *Alkalibaculum* | 0.011251786 | 0.054967753 | 0.02200968 | 0.029409739 |
| *Caproiciproducens* | 0.013414828 | 0.052923323 | 0.008919106 | 0.025085752 |
| *Paenisporosarcina* | 0.030302429 | 0.01317287 | 0.01562572 | 0.01970034 |
| *unclassified* | 0.017701222 | 0.021181975 | 0.015510486 | 0.018131228 |
| *Gelria* | 0.013394983 | 0.010980905 | 0.018183913 | 0.0141866 |
| *Syntrophomonas* | 0.01248214 | 0.015280529 | 0.011938235 | 0.013233635 |
| *Syntrophaceticus* | 0.015180981 | 0.007166041 | 0.014749942 | 0.012365655 |
| *Solibacillus* | 0.006608192 | 0.013594402 | 0.008573404 | 0.009591999 |
| *Caldicoprobacter* | 0.005715193 | 0.015196223 | 0.007490205 | 0.009467207 |
| *Planomicrobium* | 0.009326877 | 0.007924799 | 0.005024199 | 0.007425292 |
| *Clostridium* | 0.003016352 | 0.008999705 | 0.009633556 | 0.007216538 |
| *Carnobacterium* | 0.006647881 | 0.004615774 | 0.000553123 | 0.003938926 |
| *Ruminiclostridium* | 0.003929195 | 0.00383594 | 0.003433971 | 0.003733035 |
| *Ruminococcaceae* | 0.002639308 | 0.003878093 | 0.003457018 | 0.003324806 |
| *Tepidanaerobacter* | 0.002857596 | 0.001728281 | 0.0025121 | 0.002365992 |
| *Desemzia* | 0.00121051 | 0.004489314 | 0.00064531 | 0.002115045 |
| *Garciella* | 0.002956819 | 0.001665051 | 0.001521088 | 0.002047652 |
| *Lutispora* | 0.001448643 | 0.003056106 | 0.001636322 | 0.002047023 |
| *Mobilitalea* | 0.000694555 | 0.0029718 | 0.002327725 | 0.001998026 |
| *[Eubacterium]_fissicatena_group* | 0.001230354 | 0.00284534 | 0.001129292 | 0.001734995 |
| *Tissierella* | 0.002004286 | 0.001728281 | 0.001428901 | 0.001720489 |
| *Tepidimicrobium* | 0.000873154 | 0.003393331 | 0.000829684 | 0.001698723 |
| *Christensenellaceae_R-7_group* | 0.001428798 | 0.002529191 | 0.001083199 | 0.001680396 |
| *Dethiobacter* | 0.00152802 | 0.001011676 | 0.001728509 | 0.001422735 |
| *Pelotomaculum* | 0.001607398 | 0.000674451 | 0.00135976 | 0.00121387 |
| *Sporosarcina* | 0.001508176 | 0.001074906 | 0.000967965 | 0.001183682 |
| *Proteiniborus* | 0.001369265 | 0.000758757 | 0.001175386 | 0.001101136 |
| *Hazenella* | 0.000754088 | 0.001391055 | 0.00071445 | 0.000953198 |
| *Haloplasma* | 0.00085331 | 0.001011676 | 0.00071445 | 0.000859812 |
| *Bacillus* | 0.00049611 | 0.001454285 | 0.000437889 | 0.000796095 |
| *Others* | 0.00067471 | 0.000716604 | 0.000691404 | 0.000694239 |
| *Anaerosalibacter* | 0.000456422 | 0.001138136 | 0.000437889 | 0.000677482 |
| *Corynebacterium_1* | 0.000337355 | 0.001433208 | 0.000253515 | 0.000674693 |
| *Anaerosporobacter* | 0.000535799 | 0.000843064 | 0.000553123 | 0.000643995 |
| *Gracilibacter* | 0.000317511 | 0.000758757 | 0.00057617 | 0.000550813 |
| *Microbispora* | 0.000119067 | 0.001095983 | 0.000322655 | 0.000512568 |
| *Micromonospora* | 5.95333E-05 | 0.001243519 | 0.000161327 | 0.000488127 |
| *Desulfitibacter* | 0.000555644 | 0.000358302 | 0.000391795 | 0.000435247 |
| *Rummeliibacillus* | 0.000377044 | 0.000674451 | 0.000253515 | 0.000435003 |
| *Desulfotomaculum* | 0.000297666 | 0.000421532 | 0.000507029 | 0.000408742 |
| *Candidatus_Soleaferrea* | 0.000277822 | 0.000463685 | 0.000322655 | 0.000354721 |
| *Lactobacillus* | 9.92221E-05 | 0.000189689 | 0.00064531 | 0.000311407 |
| *Trichococcus* | 0.000277822 | 0.000653374 | 0 | 0.000310399 |
| *Brevibacillus* | 0.000119067 | 0.000421532 | 0.000322655 | 0.000287751 |
| *Lysinibacillus* | 3.96888E-05 | 0.000611221 | 0.000207421 | 0.00028611 |
| *Oceanobacillus* | 0.00049611 | 0.000147536 | 0.000207421 | 0.000283689 |
| *Thermopolyspora* | 0.0001786 | 0.000505838 | 0.000138281 | 0.00027424 |
| *Caloribacterium* | 3.96888E-05 | 0.000674451 | 4.60936E-05 | 0.000253411 |
| *[Eubacterium]_coprostanoligenes_group* | 0.000377044 | 0.000105383 | 0.000230468 | 0.000237632 |
| *Planifilum* | 0.000138911 | 0.000442608 | 0.000115234 | 0.000232251 |
| *Desulfosporosinus* | 0.000277822 | 0.000273996 | 9.21871E-05 | 0.000214668 |
| *Symbiobacterium* | 7.93777E-05 | 0.000189689 | 0.000368749 | 0.000212605 |
| *Laceyella* | 5.95333E-05 | 0.000189689 | 0.000299608 | 0.000182944 |
| *Jeotgalicoccus* | 0.000257977 | 0.000147536 | 0.000138281 | 0.000181265 |
| *Sporobacter* | 0.000138911 | 0.00012646 | 0.000276561 | 0.000180644 |
| *Glutamicibacter* | 5.95333E-05 | 0.00012646 | 0.000299608 | 0.000161867 |
| *Gulosibacter* | 0 | 0.000379379 | 6.91404E-05 | 0.000149506 |
| *Globicatella* | 0.000158755 | 0.000168613 | 0.000115234 | 0.000147534 |
| *Sporanaerobacter* | 0.000238133 | 0.00012646 | 6.91404E-05 | 0.000144578 |
| *Psychrobacter* | 0.000138911 | 8.43064E-05 | 0.000115234 | 0.000112817 |
| *Thermoflavimicrobium* | 5.95333E-05 | 0.000168613 | 9.21871E-05 | 0.000106778 |
| *Anaerofustis* | 0.000138911 | 8.43064E-05 | 9.21871E-05 | 0.000105135 |
| *Paenibacillus* | 3.96888E-05 | 0.000210766 | 4.60936E-05 | 9.88494E-05 |
| *Sporotomaculum* | 1.98444E-05 | 6.32298E-05 | 0.000207421 | 9.68318E-05 |
| *Aneurinibacillus* | 3.96888E-05 | 0.000168613 | 6.91404E-05 | 9.24806E-05 |
| *Thermoactinomyces* | 9.92221E-05 | 8.43064E-05 | 9.21871E-05 | 9.19052E-05 |
| *Oxobacter* | 7.93777E-05 | 0.000147536 | 4.60936E-05 | 9.10025E-05 |
| *Cryptanaerobacter* | 3.96888E-05 | 8.43064E-05 | 0.000115234 | 7.9743E-05 |
| *Geobacillus* | 5.95333E-05 | 8.43064E-05 | 6.91404E-05 | 7.09933E-05 |
| *Streptococcus* | 0 | 0.000189689 | 2.30468E-05 | 7.0912E-05 |
| *Pseudomonas* | 0 | 0.000147536 | 2.30468E-05 | 5.6861E-05 |
| *[Eubacterium]_nodatum_group* | 1.98444E-05 | 6.32298E-05 | 6.91404E-05 | 5.07382E-05 |
| *Lachnoclostridium* | 1.98444E-05 | 8.43064E-05 | 2.30468E-05 | 4.23992E-05 |
| *Methylobacter* | 3.96888E-05 | 2.10766E-05 | 4.60936E-05 | 3.56197E-05 |
| *Oligella* | 0 | 0 | 9.21871E-05 | 3.0729E-05 |
| *Papillibacter* | 1.98444E-05 | 0 | 6.91404E-05 | 2.96616E-05 |
| *Aerococcus* | 0 | 4.21532E-05 | 4.60936E-05 | 2.94156E-05 |
| *Mesorhizobium* | 0 | 4.21532E-05 | 4.60936E-05 | 2.94156E-05 |
| *Ralstonia* | 0 | 4.21532E-05 | 4.60936E-05 | 2.94156E-05 |
| *Sporobacterium* | 0 | 4.21532E-05 | 4.60936E-05 | 2.94156E-05 |
| *Dehalobacter* | 1.98444E-05 | 2.10766E-05 | 4.60936E-05 | 2.90049E-05 |
| *Hydrogenoanaerobacterium* | 1.98444E-05 | 2.10766E-05 | 4.60936E-05 | 2.90049E-05 |
| *Methylocystis* | 0 | 6.32298E-05 | 2.30468E-05 | 2.87589E-05 |
| *Microbacterium* | 3.96888E-05 | 0 | 4.60936E-05 | 2.85941E-05 |
| *Desulfurispora* | 0 | 8.43064E-05 | 0 | 2.81021E-05 |
| *Pseudoxanthomonas* | 0 | 8.43064E-05 | 0 | 2.81021E-05 |
| *Thermobacillus* | 0 | 8.43064E-05 | 0 | 2.81021E-05 |
| *Anaerotruncus* | 3.96888E-05 | 2.10766E-05 | 2.30468E-05 | 2.79374E-05 |
| *Devosia* | 3.96888E-05 | 2.10766E-05 | 2.30468E-05 | 2.79374E-05 |
| *Aminobacterium* | 5.95333E-05 | 0 | 2.30468E-05 | 2.75267E-05 |
| *Alkaliphilus* | 0 | 4.21532E-05 | 2.30468E-05 | 2.17333E-05 |
| *Nannocystis* | 1.98444E-05 | 2.10766E-05 | 2.30468E-05 | 2.13226E-05 |
| *Peptoclostridium* | 0 | 6.32298E-05 | 0 | 2.10766E-05 |
| *Salinispora* | 0 | 6.32298E-05 | 0 | 2.10766E-05 |
| *[Eubacterium]_brachy_group* | 1.98444E-05 | 4.21532E-05 | 0 | 2.06659E-05 |
| *Fonticella* | 1.98444E-05 | 4.21532E-05 | 0 | 2.06659E-05 |
| *Hyphomicrobium* | 1.98444E-05 | 4.21532E-05 | 0 | 2.06659E-05 |
| *Risungbinella* | 1.98444E-05 | 4.21532E-05 | 0 | 2.06659E-05 |
| *Phaselicystis* | 1.98444E-05 | 0 | 2.30468E-05 | 1.42971E-05 |
| *Romboutsia* | 1.98444E-05 | 0 | 2.30468E-05 | 1.42971E-05 |
| *Actinomadura* | 0 | 4.21532E-05 | 0 | 1.40511E-05 |
| *Sorangium* | 0 | 4.21532E-05 | 0 | 1.40511E-05 |
| *Aeromonas* | 1.98444E-05 | 2.10766E-05 | 0 | 1.36403E-05 |
| *Rhizobium* | 1.98444E-05 | 2.10766E-05 | 0 | 1.36403E-05 |
| *Brevibacterium* | 3.96888E-05 | 0 | 0 | 1.32296E-05 |
| *Corynebacterium glutamicum* | 0 | 0 | 2.30468E-05 | 7.68226E-06 |
| *Candidatus_Alysiosphaera* | 0 | 0 | 2.30468E-05 | 7.68226E-06 |
| *Sphingobacterium* | 0 | 0 | 2.30468E-05 | 7.68226E-06 |
| *Advenella* | 0 | 2.10766E-05 | 0 | 7.02553E-06 |
| *Gemmatimonas* | 0 | 2.10766E-05 | 0 | 7.02553E-06 |
| *Iamia* | 0 | 2.10766E-05 | 0 | 7.02553E-06 |
| *Kroppenstedtia* | 0 | 2.10766E-05 | 0 | 7.02553E-06 |
| *Luteimonas* | 0 | 2.10766E-05 | 0 | 7.02553E-06 |
| *Nocardia* | 0 | 2.10766E-05 | 0 | 7.02553E-06 |
| *Propionibacterium* | 0 | 2.10766E-05 | 0 | 7.02553E-06 |
| *Pseudonocardia* | 0 | 2.10766E-05 | 0 | 7.02553E-06 |
| *Saccharopolyspora* | 0 | 2.10766E-05 | 0 | 7.02553E-06 |
| *Shimazuella* | 0 | 2.10766E-05 | 0 | 7.02553E-06 |
| *Streptomyces* | 0 | 2.10766E-05 | 0 | 7.02553E-06 |
| *Atopobium* | 1.98444E-05 | 0 | 0 | 6.61481E-06 |
| *Chryseolinea* | 1.98444E-05 | 0 | 0 | 6.61481E-06 |
| *Erythrobacter* | 1.98444E-05 | 0 | 0 | 6.61481E-06 |
| *Exiguobacterium* | 1.98444E-05 | 0 | 0 | 6.61481E-06 |
| *Paracoccus* | 1.98444E-05 | 0 | 0 | 6.61481E-06 |
| *Salinicoccus* | 1.98444E-05 | 0 | 0 | 6.61481E-06 |
| *Sphingopyxis* | 1.98444E-05 | 0 | 0 | 6.61481E-06 |

Table S6. The pure-culture bacteria in pit muds

| NO | Strain NO | Name | Similarity | Culture Condition | Accession Number |
| --- | --- | --- | --- | --- | --- |
| 1 | NA17 | *Acidipropionibacterium acidipropionici* | 99.93 | 1 | MT269503 |
| 2 | I45 | *Acinetobacter albensis* | 98.79 | 11 | MT378378 |
| 3 | I17 | *Advenella kashmirensis* | 99.86 | 11 | MT269513 |
| 4 | F6AC | *Advenella kashmirensis* | 99.79 | 8 | MT269512 |
| 5 | H1 | *Aerococcus urinaeequi* | 99.93 | 10 | MT269520 |
| 6 | H5 | *Aerococcus urinaeequi* | 99.72 | 10 | MT269539 |
| 7 | H2 | *Aerococcus urinaeequi* | 99.86 | 10 | MT269523 |
| 8 | H3 | *Aerococcus urinaeequi* | 100.00 | 10 | MT269524 |
| 9 | H4 | *Aerococcus urinaeequi* | 99.86 | 10 | MT269534 |
| 10 | H10 | *Aerococcus urinaeequi* | 99.93 | 10 | MT269541 |
| 11 | NG9 | *Arthrobacter stackebrandtii* | 90.00 | 2 | MT269572 |
| 12 | NG3 | *Arthrobacter stackebrandtii* | 99.07 | 2 | MT269547 |
| 13 | NF33 | *Arthrobacter stackebrandtii* | 99.07 | 3 | MT269543 |
| 14 | NF46 | *Arthrobacter stackebrandtii* | 99.07 | 3 | MT269545 |
| 15 | F12 | *Bacillus coagulans* | 99.93 | 13 | MT269642 |
| 16 | F13 | *Bacillus coagulans* | 99.51 | 13 | MT269658 |
| 17 | F16 | *Bacillus coagulans* | 99.93 | 13 | MT270439 |
| 18 | K5 | *Bacillus coagulans* | 99.93 | 21 | MT269665 |
| 19 | K8 | *Bacillus coagulans* | 99.58 | 21 | MT269768 |
| 20 | K9 | *Bacillus coagulans* | 99.93 | 21 | MT269767 |
| 21 | KB1 | *Bacillus coagulans* | 99.31 | 23 | MT269771 |
| 22 | KB14 | *Bacillus coagulans* | 99.44 | 23 | MT269804 |
| 23 | KB19 | *Bacillus coagulans* | 99.93 | 23 | MT269805 |
| 24 | KB23 | *Bacillus coagulans* | 99.93 | 23 | MT269817 |
| 25 | KB25 | *Bacillus coagulans* | 100.00 | 23 | MT269875 |
| 26 | KB4 | *Bacillus coagulans* | 99.31 | 23 | MT269773 |
| 27 | KB7 | *Bacillus coagulans* | 99.52 | 23 | MT269790 |
| 28 | KB8 | *Bacillus coagulans* | 99.93 | 23 | MT269793 |
| 29 | LG10 | *Bacillus coagulans* | 99.52 | 27 | MT271937 |
| 30 | LG2 | *Bacillus coagulans* | 99.58 | 27 | MT270228 |
| 31 | LG28 | *Bacillus coagulans* | 99.51 | 27 | MT271938 |
| 32 | LG3 | *Bacillus coagulans* | 99.93 | 27 | MT270414 |
| 33 | LG8 | *Bacillus coagulans* | 100.00 | 27 | MT270417 |
| 34 | LG9 | *Bacillus coagulans* | 99.24 | 27 | MT272110 |
| 35 | F12B | *Bacillus coagulans* | 99.24 | 13 | MT269649 |
| 36 | H6 | *Bacillus coagulans* | 99.93 | 14 | MT192605 |
| 37 | K1 | *Bacillus coagulans* | 99.86 | 21 | MT192604 |
| 38 | K2 | *Bacillus coagulans* | 99.86 | 21 | MT192607 |
| 39 | K3 | *Bacillus coagulans* | 99.86 | 21 | MT192608 |
| 40 | K6 | *Bacillus coagulans* | 99.93 | 21 | MT192609 |
| 41 | K7 | *Bacillus coagulans* | 99.86 | 21 | MT192610 |
| 42 | KB15 | *Bacillus coagulans* | 99.51 | 23 | MT192614 |
| 43 | KB16 | *Bacillus coagulans* | 99.85 | 23 | MT192616 |
| 44 | KB21 | *Bacillus coagulans* | 99.86 | 23 | MT192619 |
| 45 | KB22 | *Bacillus coagulans* | 99.86 | 23 | MT192620 |
| 46 | KB6 | *Bacillus coagulans* | 99.58 | 23 | MT192612 |
| 47 | KB9 | *Bacillus coagulans* | 99.93 | 23 | MT192613 |
| 48 | LG11 | *Bacillus coagulans* | 99.79 | 27 | MT192639 |
| 49 | LG7 | *Bacillus coagulans* | 99.79 | 27 | MT192640 |
| 50 | FJ4R/NC32A | *Bacillus xiapuensis* | 97.54 | 4 | MT192642 |
| 51 | NC35B | *Bacillus foraminis* | 98.43 | 4 | MT192641 |
| 52 | KB18 | *Bacillus fumarioli* | 98.15 | 23 | MT192647 |
| 53 | KB10 | *Bacillus fumarioli* | 97.58 | 23 | MT192644 |
| 54 | NC18 | *Bacillus mesonae* | 99.71 | 4 | MT192655 |
| 55 | F11B | *Bacillus novalis* | 97.18 | 13 | MT192654 |
| 56 | KB26 | *Bacillus novalis* | 97.26 | 23 | MT192656 |
| 57 | KB27B | *Bacillus novalis* | 97.26 | 23 | MT192657 |
| 58 | NFAA | *Bacillus subtilis* | 100.00 | 3 | MT192659 |
| 59 | NC6 | *Bacillus thruingiensis* | 99.93 | 4 | MT193294 |
| 60 | JB10 | *Bacillus thuringiensis* | 100.00 | 18 | MT193295 |
| 61 | JB28 | *Bacillus thuringiensis* | 99.79 | 18 | MT193296 |
| 62 | KB11 | *Bacillus fumarioli* | 97.93 | 23 | MT192645 |
| 63 | JA48 | *Bacteroides xylanolyticus* | 99.16 | 17 | MT192666 |
| 64 | JB43 | *Bacteroides xylanolyticus* | 99.16 | 18 | MT192665 |
| 65 | NC35A | *Brevibacterium aurantiacum* | 98.50 | 4 | MT192667 |
| 66 | A1 | *Carnobacterium maltaromaticum* | 99.79 | 1 | MT192710 |
| 67 | A11 | *Carnobacterium maltaromaticum* | 99.72 | 1 | MT192709 |
| 68 | I25 | *Carnobacterium maltaromaticum* | 100.00 | 11 | MT192713 |
| 69 | A3 | *Carnobacterium maltaromaticum* | 100.00 | 7 | MT192714 |
| 70 | NA19 | *Cellulosimicrobium funkei* | 99.71 | 1 | MT192719 |
| 71 | I32A | *Cellulosimicrobium funkei* | 99.64 | 11 | MT192716 |
| 72 | I31A | *Cellulosimicrobium funkei* | 99.64 | 11 | MT264773 |
| 73 | JB12 | *Clostridium amygdalinum* | 99.86 | 18 | MT264792 |
| 74 | JB20 | *Clostridium amygdalinum* | 99.79 | 18 | MT264911 |
| 75 | JB4 | *Clostridium amygdalinum* | 99.72 | 18 | MT264780 |
| 76 | JB5 | *Clostridium amygdalinum* | 97.73 | 18 | MT264779 |
| 77 | JB9 | *Clostridium amygdalinum* | 99.86 | 18 | MT269005 |
| 78 | JA66 | *Clostridium amylolyticum* | 100.00 | 17 | MT264909 |
| 79 | F14 | *Clostridium celerecrescens* | 99.57 | 13 | MT264945 |
| 80 | JA21 | *Clostridium celerecrescens* | 99.85 | 17 | MT264946 |
| 81 | JA22 | *Clostridium celerecrescens* | 99.29 | 17 | MT264964 |
| 82 | JA24 | *Clostridium celerecrescens* | 99.71 | 17 | MT264994 |
| 83 | JA40 | *Clostridium celerecrescens* | 99.30 | 17 | MT264995 |
| 84 | JA30 | *Clostridium indolis* | 95.85 | 17 | MT265054 |
| 85 | JA42 | *Clostridium indolis* | 99.71 | 17 | MT265058 |
| 86 | JA52 | *Clostridium indolis* | 99.86 | 17 | MT265057 |
| 87 | JA81 | *Clostridium indolis* | 95.43 | 17 | MT265071 |
| 88 | JA17B | *Clostridium liquoris* | 99.42 | 17 | MT265072 |
| 89 | JA3 | *Clostridium luticellarii* | 97.05 | 17 | MT265070 |
| 90 | JA19 | *Clostridium celerecrescens* | 99.50 | 17 | MT380744 |
| 91 | LG1 | *Clostridium saccharobutylicum* | 99.78 | 26 | MT265247 |
| 92 | F12B | *Clostridium sphenoides* | 99.57 | 13 | MT265326 |
| 93 | FH14A | *Clostridium sphenoides* | 99.36 | 13 | MT265385 |
| 94 | J9 | *Clostridium sphenoides* | 99.37 | 16 | MT265384 |
| 95 | JA44 | *Clostridium sphenoides* | 95.81 | 17 | MT265388 |
| 96 | JA47 | *Clostridium sphenoides* | 99.64 | 17 | MT265393 |
| 97 | F12A | *Clostridium sphenoides* | 99.30 | 13 | MT265303 |
| 98 | F14A | *Clostridium sphenoides* | 99.30 | 13 | MT265327 |
| 99 | JA33 | *Clostridium sphenoides* | 97.61 | 17 | MT265387 |
| 100 | JA39 | *Clostridium sphenoides* | 99.70 | 17 | MT265389 |
| 101 | JA58 | *Clostridium sphenoides* | 95.91 | 17 | MT265394 |
| 102 | JA61 | *Clostridium sphenoides* | 99.57 | 17 | MT265677 |
| 103 | JA75 | *Clostridium sphenoides* | 95.73 | 17 | MT265676 |
| 104 | JA87 | *Clostridium sphenoides* | 95.80 | 17 | MT265803 |
| 105 | JA95 | *Clostridium sphenoides* | 99.63 | 17 | MT265679 |
| 106 | JB38 | *Clostridium sphenoides* | 99.64 | 18 | MT266834 |
| 107 | JB41 | *Clostridium sphenoides* | 99.50 | 18 | MT266831 |
| 108 | JB45 | *Clostridium sphenoides* | 99.30 | 18 | MT263175 |
| 109 | JB46 | *Clostridium sphenoides* | 99.30 | 18 | MT263698 |
| 110 | JB47 | *Clostridium sphenoides* | 99.56 | 18 | MT263722 |
| 111 | JB49 | *Clostridium sphenoides* | 99.64 | 18 | MT263730 |
| 112 | JBA8 | *Clostridium sphenoides* | 99.16 | 18 | MT263750 |
| 113 | KB19 | *Clostridium sphenoides* | 99.42 | 23 | MT263949 |
| 114 | JB67 | *Clostridium swellfunianum* | 95.66 | 18 | MT263975 |
| 115 | C28 | *Clostridium tyrobutyricum* | 99.93 | 12 | MT263977 |
| 116 | F18 | *Clostridium tyrobutyricum* | 100.00 | 13 | MT263987 |
| 117 | H2 | *Clostridium tyrobutyricum* | 99.93 | 14 | MT264778 |
| 118 | H4 | *Clostridium tyrobutyricum* | 100.00 | 14 | MT264793 |
| 119 | H5 | *Clostridium tyrobutyricum* | 100.00 | 14 | MT264908 |
| 120 | JA17A | *Clostridium tyrobutyricum* | 100.00 | 17 | MT264963 |
| 121 | JA41 | *Clostridium tyrobutyricum* | 100.00 | 17 | MT265031 |
| 122 | JA67 | *Clostridium tyrobutyricum* | 100.00 | 17 | MT265060 |
| 123 | LG12 | *Clostridium tyrobutyricum* | 100.00 | 27 | MT265221 |
| 124 | LG24 | *Clostridium tyrobutyricum* | 100.00 | 27 | MT269011 |
| 125 | LG5 | *Clostridium tyrobutyricum* | 100.00 | 27 | MT265080 |
| 126 | JA20 | *Clostridium tyrobutyricum* | 100.00 | 17 | MT264993 |
| 127 | I15B | *Corynebacterium glutamicum* | 99.85 | 11 | MT266923 |
| 128 | NG12 | *Delftia lacustris* | 100.00 | 2 | MT266922 |
| 129 | NC16 | *Delftia lacustris* | 100.00 | 4 | MT266832 |
| 130 | NC46 | *Delftia lacustris* | 100.00 | 4 | MT269010 |
| 131 | NC15 | *Delftia lacustris* | 100.00 | 4 | MT266833 |
| 132 | I7 | *Dietzia maris* | 99.85 | 11 | MT266927 |
| 133 | NF27 | *Enterococcus crotali* | 99.93 | 3 | MT266930 |
| 134 | NF45 | *Enterococcus crotali* | 99.58 | 3 | MT266953 |
| 135 | NA27 | *Enterococcus crotali* | 99.66 | 1 | MT266928 |
| 136 | NA11 | *Enterococcus crotali* | 99.86 | 1 | MT266929 |
| 137 | JA37 | *Fusicatenibacter saccharivorans* | 92-93 | 17 | MT266983 |
| 138 | JA46B | *Fusicatenibacter saccharivorans* | 92-93 | 17 | MT267295 |
| 139 | JA65 | *Fusicatenibacter saccharivorans* | 92-93 | 17 | MT267296 |
| 140 | JA70 | *Fusicatenibacter saccharivorans* | 92-93 | 17 | MT267335 |
| 141 | JA73 | *Fusicatenibacter saccharivorans* | 92-93 | 17 | MT267338 |
| 142 | JB37 | *Clostridium sphenoides* | 99.57 | 18 | MT263291 |
| 143 | F5A | *Glutamicibacter creatinolyticus* | 99.86 | 8 | MT267336 |
| 144 | F6AA | *Glutamicibacter creatinolyticus* | 99.93 | 8 | MT235216 |
| 145 | F6BA | *Glutamicibacter creatinolyticus* | 99.93 | 8 | MT235529 |
| 146 | FJ3R/NC32A | *Gordonia terrae* | 100.00 | 4 | MT262510 |
| 147 | NA16 | *Gulosibacter molinativorax* | 99.93 | 1 | MT262511 |
| 148 | NF28 | *Kocuria carniphila* | 99.64 | 3 | MT262515 |
| 149 | Y5 | *Lactococcus lactis subsp. cremoris* | 100.00 | 7 | MT262559 |
| 150 | NG2 | *Lysinibacillus sphaericus* | 99.93 | 2 | MT262592 |
| 151 | C1B | *Lysinibacillus sphaericus* | 99.93 | 9 | MT262593 |
| 152 | NG22 | *Methylobacterium aminovorans* | 99.85 | 2 | MT262874 |
| 153 | NC8 | *Microbacterium schleiferi* | 99.27 | 4 | MT262894 |
| 154 | NC28 | *Microbacterium hydrocarbonoxydans* | 99.79 | 4 | MT262875 |
| 155 | NA14A | *Microbacterium oxydans* | 99.93 | 1 | MT262881 |
| 156 | NA14B | *Microbacterium oxydans* | 100.00 | 1 | MT262880 |
| 157 | JA59 | *Muricomes intestini* | 99.85 | 17 | MT262895 |
| 158 | JA82 | *Muricomes intestini* | 99.93 | 17 | MT262991 |
| 159 | JA88 | *Muricomes intestini* | 99.71 | 17 | MT262992 |
| 160 | JA69 | *Muricomes intestini* | 99.79 | 17 | MT262925 |
| 161 | JA76 | *Muricomes intestini* | 99.78 | 17 | MT262917 |
| 162 | JB61 | *Paenibacillus faecis* | 99.72 | 18 | MT263033 |
| 163 | JB62 | *Paenibacillus faecis* | 99.79 | 18 | MT263031 |
| 164 | C3BA | *Paenibacillus glucanolyticus* | 100.00 | 9 | MT263032 |
| 165 | I46 | *Paenibacillus glucanolyticus* | 100.00 | 11 | MT263071 |
| 166 | I26 | *Paraburkholderia fungorum* | 99.93 | 11 | MT263072 |
| 167 | H1A | *Paracoccus marcusii* | 100.00 | 10 | MT263076 |
| 168 | C10 | *Paracoccus versutus* | 99.92 | 9 | MT263174 |
| 169 | NF4 | *Planococcus rifietoensis* | 99.52 | 3 | MT263532 |
| 170 | NF11 | *Planococcus rifietoensis* | 99.79 | 3 | MT267361 |
| 171 | NF4A | *Planococcus rifietoensis* | 100.00 | 3 | MT267355 |
| 172 | NF29 | *Planococcus rifietoensis* | 99.50 | 3 | MT269280 |
| 173 | NF36 | *Planomicrobium flavidum* | 98.38 | 3 | MT269283 |
| 174 | NC1 | *Planomicrobium glaciei* | 99.20 | 4 | MT269277 |
| 175 | NF22A | *Planomicrobium okeanokoites* | 99.93 | 3 | MT269279 |
| 176 | NF22B | *Planomicrobium okeanokoites* | 99.86 | 3 | MT269281 |
| 177 | NC34A | *Planomicrobium okeanokoites* | 98.72 | 4 | MT269282 |
| 178 | NC11 | *Pseudoxanthomonas indica* | 98.98 | 4 | MT269284 |
| 179 | NC10 | *Pseudoxanthomonas indica* | 99.19 | 4 | MT269278 |
| 180 | NC19 | *Pseudoxanthomonas indica* | 99.19 | 4 | MT269546 |
| 181 | NC23 | *Pseudoxanthomonas indica* | 99.19 | 4 | MT269573 |
| 182 | NC24 | *Pseudoxanthomonas indica* | 99.19 | 4 | MT269579 |
| 183 | NC12 | *Pseudoxanthomonas indica* | 99.11 | 4 | MT269544 |
| 184 | NC7 | *Psychrobacter faecalis* | 99.79 | 4 | MT269580 |
| 185 | JA28 | *Rummeliibacillus suwonensis* | 99.64 | 17 | MT269581 |
| 186 | WTY | *Sphingomonas aquatilis* | 100.00 | 11 | MT269590 |
| 187 | I16A | *Sphingomonas aquatilis* | 97.94 | 11 | MT269582 |
| 188 | I35 | *Sphingomonas aquatilis* | 97.86 | 11 | MT269583 |
| 189 | I40 | *Sphingomonas aquatilis* | 98.27 | 11 | MT269584 |
| 190 | I42 | *Sphingomonas aquatilis* | 98.06 | 11 | MT269586 |
| 191 | I44 | *Sphingomonas aquatilis* | 97.86 | 11 | MT269587 |
| 192 | I45A | *Sphingomonas aquatilis* | 98.00 | 11 | MT269588 |
| 193 | NF25 | *Sphingopyxis chilensis* | 99.11 | 3 | MT269589 |
| 194 | NF8 | *Sporosarcina koreensis* | 99.58 | 3 | MT269643 |
| 195 | NF26 | *Sporosarcina psychrophila* | 98.90 | 3 | MT269646 |
| 196 | NF7 | *Sporosarcina globispora* | 98.12 | 3 | MT269644 |
| 197 | NA3 | *Staphylococcus epidermidis* | 99.93 | 1 | MT269645 |
| 198 | NA4 | *Staphylococcus epidermidis* | 99.93 | 1 | MT270438 |
| 199 | NFBB | *Staphylococcus warneri* | 100.00 | 3 | MT269647 |
| 200 | F4B | *Stenotrophomonas maltophilia* | 99.79 | 8 | MT269663 |
| 201 | F6BB | *Stenotrophomonas maltophilia* | 99.79 | 8 | MT269662 |
| 202 | I31BB | *Stenotrophomonas maltophilia* | 99.86 | 11 | MT269664 |
| 203 | NC4 | *Stenotrophomonas bentonitica* | 99.64 | 4 | MT269648 |
| 204 | NG7A | *Stenotrophomonas bentonitica* | 99.64 | 2 | MT269655 |
| 205 | NF12 | *Stenotrophomonas bentonitica* | 99.72 | 3 | MT269656 |
| 206 | NF1 | *Streptococcus parauberis* | 99.79 | 3 | MT269770 |
| 207 | NF9 | *Streptococcus parauberis* | 99.37 | 3 | MT269758 |
| 208 | NF21 | *Streptococcus parauberis* | 99.79 | 3 | MT269772 |
| 209 | NF31 | *Streptococcus parauberis* | 99.93 | 3 | MT269757 |
| 210 | NF32 | *Streptococcus parauberis* | 99.79 | 3 | MT269769 |
| 211 | H8 | *Streptococcus parauberis* | 99.79 | 10 | MT269786 |
| 212 | NF6 | *Streptococcus parauberis* | 99.50 | 3 | MT269785 |
| 213 | JA46 | *Fusicatenibacter saccharivorans* | 92.68 | 17 | MT269540 |
| 214 | JA74 | *Fusicatenibacter saccharivorans* | 92.49 | 17 | MT269542 |
| 215 | NF24 | *Trichococcus flocculiformis* | 99.86 | 3 | MT269787 |
